# Pharmacological Targeting of CSF1R Inhibits Microglial Proliferation and Aggravates the Progression of Cerebral Ischemic Pathology

**DOI:** 10.3389/fncel.2020.00267

**Published:** 2020-10-16

**Authors:** Boru Hou, Cheng Jiang, Dong Wang, Gang Wang, Zening Wang, Miaojuan Zhu, Yuchen Kang, Jiacheng Su, Pengfei Wei, Haijun Ren, Furong Ju

**Affiliations:** ^1^Department of Neurosurgery, Lanzhou University Second Hospital, Lanzhou, China; ^2^Second Clinical Medical College, Lanzhou University, Lanzhou, China; ^3^Shenzhen Key Lab of Neuropsychiatric Modulation and Collaborative Innovation Center for Brain Science, CAS Center for Excellence in Brain Science, Shenzhen Institutes of Advanced Technology, Chinese Academy of Sciences, Shenzhen, China; ^4^School of Life Sciences, Lanzhou University, Lanzhou, China

**Keywords:** microglia, CSF1R, ischemia, neuron, ki20227

## Abstract

Ischemic stroke can induce rapid activation of the microglia. It has been reported that the microglia’s survival is dependent on colony-stimulating factor 1 receptor (CSF1R) signaling and that pharmacological inhibition of CSF1R leads to morphological changes in the microglia in the healthy brain. However, the impact of CSF1R inhibition on neuronal structures and motor ability after ischemia–reperfusion remains unclear. In this study, we investigated microglial de-ramification, proliferation, and activation after inhibition of CSF1R by a tyrosine kinase inhibitor (ki20227) in a mouse model of global cerebral ischemia induced by bilateral common carotid artery ligation (BCAL). In addition to microglial morphology, we evaluated the mRNA expression of cytokines, chemokines, and inflammatory receptors. Our results show that pharmacological inhibition of CSF1R in ischemic mice resulted in the blockade of microglial proliferation and a shift in microglial morphology reflected by excessive de-ramification and a more activated phenotype accompanied by an enhanced innate immune response. Furthermore, we show that pharmacological inhibition of CSF1R in ischemic mice resulted in the aggravation of neuronal degeneration and behavioral impairment. Intravital two-photon imaging revealed that although pharmacological inhibition of CSF1R did not affect the recovery of dendritic structures, it caused a significant increase in spine elimination during reperfusion in ischemic mice. These findings suggest that pharmacological inhibition of CSF1R induces a blockade of microglial proliferation and causes acute activation of the microglia accompanied by a severe inflammatory response. It aggravates neuronal degeneration, loss of dendritic spines, and behavioral deficits after transient global cerebral ischemia.

## Introduction

As the primary resident immune cells of the central nervous system (CNS), the microglia play an essential role in physiology and CNS pathologies such as stroke (Kettenmann et al., 2011; Zhan et al., 2014). Importantly, the microglia exhibit changes in morphology and gene expression, particularly in response to stroke (Morrison and Filosa, 2013), and microglial activation is correlated with de-ramification and attenuated process motility after stroke (Orr et al., 2009). Various studies have shown that the microglia continuously survey the cerebral microenvironment with their extensive processes (Davalos et al., 2005; Nimmerjahn et al., 2005), interact with neurons, and regulate the turnover of synaptic structures (Winship and Murphy, 2008; Wake et al., 2009, 2013; Jolivel et al., 2015). Recent studies have shown that microglia positively contribute to normal CNS physiology to allow the completion of multiple learning tasks and motor learning-dependent synapse formation through brain-derived neurotrophic factor (BDNF) signaling (Parkhurst et al., 2013). Besides, two-photon imaging studies have directly demonstrated microglia-induced spine formation in the developing somatosensory cortex *in vivo* (Miyamoto et al., 2016). Activated microglia remove damaged neural cells, cellular debris, and dysfunctional synapses and produce pro- or anti-inflammatory factors after stroke (Turrin and Rivest, 2006; Denes et al., 2010; Iadecola and Anrather, 2011; Vinet et al., 2012; Prinz and Priller, 2014; Masuch et al., 2016). Although there have been many reports on the characteristics of the microglia under both physiological and pathological conditions in the past decades, little is known about whether the recovery of neurons is closely associated with the microglia during the pathological process of stroke. Studying the functional role of the microglia in shaping complex neuronal networks after stroke is difficult since strategies that target the microglia *in vivo* also affect other macrophages. Recently, inhibition of colony-stimulating factor 1 receptor (CSF1R) on the microglia, loss of colony-stimulating factor 1 (CSF-1), and genetic knockout of CSF1R were shown to reduce the population of normal microglia *in vivo* (Li et al., 2006; Erblich et al., 2011; Ginhoux et al., 2014), providing strategies for further research on the microglia. Depletion of the microglia not only causes defects in neuronal structure and function in healthy mice, affecting performance on multiple learning tasks, but also increases the infarct size and causes deregulation of neuronal calcium responses in cerebral ischemia (Parkhurst et al., 2013; Szalay et al., 2016). In addition, some studies suggest that microglia depletion by long-term treatment with a CSF1R inhibitor increases the numbers of neutrophils and the size of the ischemic lesion (Szalay et al., 2016; Otxoa-De-Amezaga et al., 2019), but some other study has indicated that eliminating the microglia does not improve neurogenesis after traumatic brain injury (Willis et al., 2020). Thus, the microglia may act as a double-edged sword in many neural diseases, and its in stroke still remains debatable. PLX3397 and PLX5622 are common and robust inhibitors of CSF1R that can cause a dramatic reduction in the microglial population in the adult brain (Elmore et al., 2014; Willis et al., 2020). GW2580 can specifically block microglial proliferation in APP/PS1 mice, and ki20227 inhibits the turnover/expansion of myeloid cells in an experimental autoimmune encephalomyelitis (EAE) animal model (Ohno et al., 2006, 2008; Uemura et al., 2008; Olmos-Alonso et al., 2016). However, neither of these inhibitors induces significant changes in the microglial survival rate (Hou et al., 2016; Olmos-Alonso et al., 2016).

In the present study, a reversible global cerebral ischemia model and *in vivo* imaging were used to determine the precise roles of the microglia in the brain after stroke. Here, we report that ki20227 treatment blocks microglial proliferation and causes excessive microglial activation after cerebral ischemia. Furthermore, ki20227 aggravates neuronal degeneration, loss of dendritic spines, and behavioral deficits after cerebral ischemia.

## Materials and Methods

### Animals

Transgenic mice (Thy1-YFP line H) expressing yellow fluorescent protein (YFP) in layer 5 pyramidal neurons and CX3CR1^GFP/+^ mice expressing green fluorescent protein (GFP) in the microglia were purchased from Jackson Laboratory. All animals were bred in the Laboratory Centre for Basic Medical Sciences, Lanzhou University. The mice had free access to food and clean water and were housed under a normal 24-h light cycle (12-h light/12-h dark) at 22 ± 2°C. Transgenic mice of both sexes aged 3–5 months and weighing 20–30 g were used in this study. All experiments in this study were carried out strictly following the rules set by the Ethics Committee of Lanzhou University, China.

### Global Ischemia Model

The method of bilateral common carotid artery ligation (BCAL), which was used to induce global ischemia, was described in previous studies (Zhang et al., 2005; Winship and Murphy, 2008). Briefly, each animal was deeply anesthetized with a mixture of 2% ketamine and 0.2% xylazine by intraperitoneal injection, and then a thinned-skull cranial window was created above the somatosensory cortex. After that, the animal was placed in the supine position, and the carotid arteries were occluded through a midline incision. To ensure that global ischemia was effectively induced in mice, reduced blood flow and beaded dendrites were confirmed by two-photon imaging through the cranial window. If decreases in blood flow and beading of dendrites were not observed within 10 min after surgery through the cranial window by intravital two-photon imaging, then global ischemia had not been successfully induced. The mice were subjected to 60 min of transient ischemia after occlusion of the common carotid arteries at room temperature by tying surgical sutures. After 60 min of bilateral common carotid artery occlusion, the surgical sutures were untied to allow reperfusion of the blood vessels. To support recovery and enhance the survival rate, we placed the mice on a heating pad after BCAL until they fully awoke.

### Antagonism of CSF1R

Inhibition of the tyrosine kinase activity of CSF1R was achieved by the administration of ki20227, and inhibition of protein kinases was evaluated based on the expression profiles, as previously described (Ohno et al., 2008). The mice were treated intragastrically with ki20227 (each mouse was given 2 mg g^−1^ day^−1^) once daily for 3 days of reperfusion. Vehicle (0.5% methylcellulose in distilled water) was administered intragastrically during reperfusion after 60 min of ischemia, and reperfusion was continued for 3 days. The mice were divided into four groups: the intragastric ki20227 administration group (ki20227-treated sham mice), vehicle group (vehicle-treated sham mice), ki20227 treatment (once daily) during reperfusion after 60 min of ischemia group (ki20227-treated stroke mice), and vehicle treatment (once daily) during reperfusion after 60 min of ischemia group (vehicle-treated stroke mice).

### Two-Photon Imaging and Data Analysis

Intravital two-photon imaging was performed as described in previous studies (Grutzendler et al., 2002; Zhang et al., 2005). Briefly, to ensure the immobility of the animals, a custom-made metal frame and a steel plate were used to fix the skulls. Then, the skulls of anesthetized mice were exposed and the region of interest, i.e., the imaging site (a 2 × 2-mm^2^ area of the skull with the center located −1.5 mm from Bregma and 2.0 mm from the midline), was marked and then thinned to a thickness of 25 μm by a high-speed dental drill. After the step distance was set to an appropriate depth for dendritic spines (0.75 μm), real-time images of the dendritic structure were acquired with a two-photon microscope with a ×25 water-immersion objective lens at zoom 4 (×25/1.05, Olympus) at a wavelength of 920 nm. Two-photon image analysis was carried out as described previously (Ju et al., 2018). To quantify the percentage of blebbed dendrites, we used a custom-designed macro to mark dendrites at a fixed length of 20 μm and calculated the total length of the selected dendritic segments. The percentage of blebbed dendrites was calculated as the total length of blebbed dendrites/total length of dendrites. To quantify blood flow, we intravenously administered Evans blue (EB) solution (10 mg/ml; 2.5 ml/kg) before surgery. Blood flow was evaluated with a two-photon microscope with a ×25 water-immersion objective lens at zoom 4 (×25/1.05, Olympus) at a wavelength of 920 nm. A line was drawn along the center axis of an arteriole (10–15 μm in diameter), and repeated line scanning was used to measure the velocity of red blood cells (RBCs). To estimate RBC velocity, we fit a line to the arcs created by moving RBCs in line scans and calculated the change in position (Δ*x*/Δ*t*). Thus, the *x*-axis presents the distance which the red blood cells have traveled; each interval between the lines aligned along the *y*-axis reflects the period between each scan. The velocity was presented as pixel distance/line time. Raw data have been extracted from the ImageJ measurement. Dividing the distance along the *x*-axis (calculated by pixels’ edge) by the time period along the *y*-axis (in seconds) can result in the velocity of blood cells (in micrometers per second). Six to 10 vessels were analyzed per mouse (*n* = 6). To quantify dendritic spine changes, the same dendritic segments were carefully imaged at different time points. More than 200 spines selected from the image stacks were counted for each mouse. Spine elimination was calculated as the number of lost spines/the number of preexisting spines, and spine formation was calculated as the number of new spines/the number of preexisting spines.

### Nissl and Immunofluorescence Staining

Brain tissues fixed with 4% paraformaldehyde (PFA) were washed in phosphate-buffered saline (PBS) and sectioned (30 μm) on a vibrating microtome (Leica). We visualized Nissl bodies in layers 1–3 of the somatosensory cortex at −1.5 to  −2.0 mm from Bregma and 2.0 mm from the midline. Nissl bodies were stained with cresyl violet. Serial brain sections were collected at 120-μm intervals. Then, the slices were treated as follows: air-dried for 15 min and immersed in 95% ethanol for 15 min, 70% ethanol for 1 min, 50% ethanol for 1 min, ddH_2_O twice for 1 min each, cresyl violet for 10 min, ddH_2_O for 1 min, 50% ethanol for 1 min, 70% ethanol for 2 min, 95% ethanol for 2 min, 100% ethanol for 2 min, and xylene for 10 min. After these procedures, the sections were sealed with neutral balsam for microscopy. All images were analyzed using ImageJ software^1^ to count the Nissl bodies. Eight to 10 somatosensory cortex slices (−1.0 to −2.5 mm from Bregma) per mouse were analyzed (*n* = 6).

To immunofluorescently label neurons, the brain sections were washed with PBS and blocked with 10% goat serum in PBS for 0.5 h. The sections were incubated with a primary antibody against NeuN (1:200; Millipore) diluted in buffer containing 0.01% Triton X-100 and 10% goat serum for 12 h at 4°C. The brain sections were incubated with rhodamine-conjugated goat anti-rat secondary antibody (1:100; ZSGB-BIO) for 1 h at room temperature. For each confocal experiment, all brain sections containing layers 1–3 of the somatosensory cortex, located −1.5 to −2.0 mm from Bregma and 2.0 mm from the midline, were imaged. All images were analyzed using ImageJ software to count the number of cells. Eight to 10 brain slices per mouse were analyzed (*n* = 6).

To identify proliferating microglia after stroke, bromodeoxyuridine (BrdU) immunofluorescence was performed as described in our preliminary studies. Briefly, BrdU (50 mg/kg; Sigma) was administered to the mice *via* intraperitoneal injection two times per day starting from the 12th hour after BCAL. After 3 days of reperfusion, the brain tissues of the mice were fixed with 4% PFA. For BrdU immunofluorescence, the brain sections were washed with PBS for 10 min, treated with 2 M HCl at 37°C for 30 min, neutralized with 0.1 M borate buffer (pH 8.5) for 3 × 15 min, and incubated overnight with a rat monoclonal antibody against BrdU (1:500; AbD Serotec). The brain tissues were incubated with rhodamine-conjugated goat anti-rat secondary antibody (1:100; ZSGB-BIO) for 1 h at room temperature. For each confocal experiment, all brain sections containing layers 1–3 of the somatosensory cortex, located −1.5 to −2.0 mm from Bregma and 2.0 mm from the midline, were imaged. All images were analyzed using ImageJ software to count the number of cells. Eight to 10 brain slices per mouse were analyzed (*n* = 6).

### Microglial Skeleton and Soma Size Analysis

We observed microglial morphology changes in layers 1–3 of the somatosensory cortex, located −1.5 to −2.0 mm from Bregma and 2.0 mm from the midline. This area was also observed *in vivo*. For microglial skeleton analysis, GFP-positive microglia extracted from CX3CR1^GFP/+^ mice were used to acquire images of microglial morphology. Brain tissues fixed with 4% PFA were washed in PBS and sectioned (60 μm) on a vibrating microtome (Leica). For cell skeletonization analysis, repeated *z-series* of about 60 optical sections [1,024 × 1,024 pixel arrays, *z-stack* at 1-μm intervals, zoom 4 (×25/1.05)] were obtained with an Olympus confocal microscope. All images were analyzed using ImageJ software. The *z-stack* of a single microglia was approximately 60 μm, and 10–15 microglia in the somatosensory cortex of each mouse were analyzed (*n* = 6). The analytic skeleton plugin^2^ was applied to collect data focused on the branch point number and the total length of the microglial process. The images were analyzed with a custom-coded macro plugin to perform skeletonized analysis (“Plugin” < “Process” < “Smooth 3D”; radius, 0.3–0.8). The file was properly converted to binary format after the final images had been stacked (“Image” < “Adjust” < “Threshold”). The skeletonization plugin (2D/3D) was used to generate skeletonized images (Lee et al., 1994). For microglial skeleton analysis, approximately 10 relatively intact microglia per mouse were analyzed (*n* = 6). For microglial soma size analysis, the soma was outlined and analyzed with ImageJ (“Polygon selections” < “Measure” < “Area”). Approximately 30 relatively intact microglial soma per mouse were analyzed (*n* = 6).

### Behavioral Tests

The open-field test was carried out as described previously to evaluate animal behavioral deficits after reperfusion for different durations (Olmos-Alonso et al., 2016). The mice were placed in individual cages (25 cm × 25 cm × 31 cm) for 10 min, and the total distance traveled (in centimeters) was measured. The rotarod test was performed on an apparatus (ZB-200 rotarod system, TME Technology) as described previously (Zhu et al., 2017a). Mice were placed on the rod and allowed to run, and the speed of the rod accelerated from 10 to 40 rpm within 300 s. The mice were subjected to three trials per day for 3 days before surgery. The mice were tested 3 days after reperfusion. The body weight and food intake of each mouse were monitored during the experiment.

### Analysis of Gene Expression by qPCR

Mice from both the ischemic and control groups were treated with vehicle or ki20227 (*n* = 6/group), and tissue samples of the somatosensory cortex (centered around the area −1.5 mm from Bregma and 2.0 mm from midline) were extracted under a dissecting microscope after transcardial perfusion with PBS. RNA was extracted with an RNA Extraction Kit (TaKaRa) and quantified with a Nanodrop spectrophotometer (Thermo Fisher Scientific), and RNA integrity was checked *via* gel (2% agarose) electrophoresis. The RNA was then reverse-transcribed with a reverse-transcription kit with gDNA Eraser (Applied Biosystems, Foster City, CA, USA). The expression of genes was analyzed *via* quantitative polymerase chain reaction (qPCR) using iTaq™ Universal SYBR^®^ Green Supermix (Bio-Rad). Glyceraldehyde-3-phosphate dehydrogenase (GAPDH) was chosen as the reference gene. The relative quantity of messenger RNAs (mRNAs) was determined by the 2^−ΔΔCT^ method. The custom-designed gene-specific primers (GENEWIZ) were as follows: CSF1 (FW: GGAACAGCTGGATGATC, RV: GAGGAGCAGAACAAGGC); BCL2 (FW: GTGCAAGTGTAAATTGCCGAG, RV: GAGACTTCTGAAGATCGATGG); CCL3 (FW: CCATATGGAGCTGACACCCC, RV: GAGCAAAGGCTGCTGGTTTC); CXCL10 (FW: CCAAGTGCTGCCGTCATTTT, RV: CTCAACACGTGGGCAGGATA); CSF1R (FW: GCAGTACCACCATCCACTTGTA, RV: GTGAGACACTGTCCTTCAGTGC); IL34 (FW: CTTTGGGAAACGAGAATTTGGAGA, RV: GCAATCCTGTAGTTGATGGGGAAG); Cd4 (FW: GTTTTCGCTACATGACTGCACA, RV: AGGTTGTCCAACTGACATCTTTC); Cd80 (FW: AGTTTCTCTTTTTCAGGTTGTGAA, RV: ACATGATGGGGAAAGCCAGG); CCR2 (FW: AGGAGCCATACCTGTAAATGCC, RV: TGTGGTGAATCCAATGCCCT); CCL19 (FW: CCTGCCTCAGATTATCTGCCA, RV: GTGACCCAGCGCCCCATCCCTGG); Ccl5 (FW: GTGCCCACGTCAAGGAGTAT, RV: TTCTCTGGGTTGGCACACAC): TNF-α (FW: GACGTGGAACTGGCAGAAGA, RV: ACTGATGAGAGGGAGGCCAT); Ccl4 (FW: GGCTCTGACCCTCCCACTTCCT, RV: CCAGTGAGCCCTGGGTCAC); Ccl2 (FW: TTAAAAACCTGGATCGGAACCAA, RV: GCATTAGCTTCAGATTTACGGGT); TGF-β (FW: GGCGATACCTCAGCAACCG, RV: CTAAGGCGAAAGCCCTCAAT); and GAPDH (FW: CGTGCCGCCTGGAGAAACCTG, RV: AGAGTGGGAGTTGCTGTTGAAGTCG). The RNA samples were derived from six pregnant mice, and at least three technical replicates of each RNA sample were analyzed by qRT-PCR.

### Statistical Analysis

SPSS software was used for all statistical analyses. Analysis of variance and two-tailed unpaired *t*-test was used to compare two groups, and one-way ANOVA was performed to compare three or more groups. Two-way ANOVA was performed to analyze the behavioral data. The results are presented as the mean ± standard error of the mean, with *p < 0.05 and **p < 0.01.

## Results

### BCAL Induces Ischemia and Alters Dendritic Structure and Blood Flow

**Figure 1:**
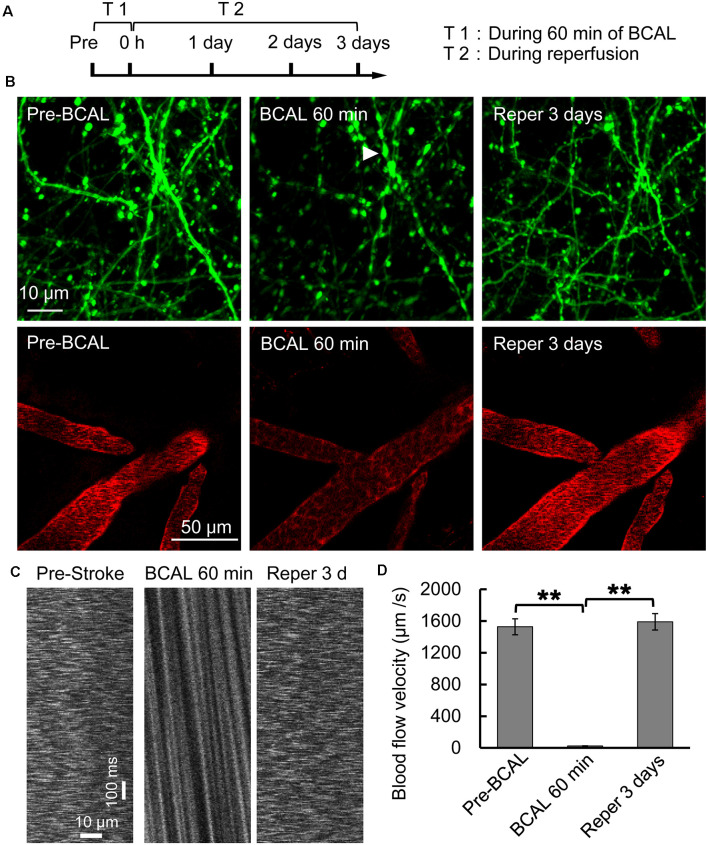
Two-photon imaging of a murine model of global ischemia. **(A)** Timeline showing the time points of tyrosine kinase inhibitor (ki20227) treatment in the transient ischemia–reperfusion model. **(B)** Two-photon images showing Evans blue (EB)-labeled blood vessels (*red*; *scale bar*, 50 μm) and dendritic structures labeled with Thy1-YFP line H (*green*; *scale bar*, 10 μm) before surgery, after 60 min of bilateral common carotid artery ligation (BCAL), and after 3 days of reperfusion. The *arrowheads* indicate blebbed dendrites. **(C)** Two-photo line-scanning imaging of the blood flow velocity before ischemia, 60 min after ischemia, and 3 days after reperfusion. *Scale bar*, 10 μm and100 ms. **(D)** Quantification of blood flow velocity. Six to 10 vessels were analyzed per mouse. *n* = 6, **p < 0.01.

Whole-brain ischemia was induced to observe changes in neurons, microglial activity, and mobility during stroke (Figure 1A). In the whole-brain ischemia model, blood flow was controlled based on the duration of common carotid artery ligation, plasma was labeled with EB by intravenous injection, and the success of the model was judged based on the slowing or stopping of blood flow (marked by red fluorescence) *via* intravital two-photon imaging (Figure 1B). Transgenic mice (Thy1-YFP line H) expressing YFP in layer 5 pyramidal neurons were used for imaging. In previous studies by our laboratory (Zhang et al., 2005; Zhu et al., 2017a), *in vivo* two-photon imaging revealed blebbed dendrites on layer 5 pyramidal neurons in the somatosensory cortex and blocked blood flow, as visualized with EB, after 60 min of BCAL (Figure 1B). Subsequently, after 3 days of reperfusion, blood flow velocity was restored to normal levels and the dendritic structure rapidly recovered [blood flow velocity (in micrometers per second): before BCAL, 1,563.9 ± 185.0; after 560 min of BCAL, 20.5 ± 6.8; after 3 days of reperfusion, 1,578.3 ± 167.1; **p < 0.01; Figures 1C,D].

### Morphological Transition of Microglia in Response to CSF1R Signaling Inhibition

**Figure 2:**
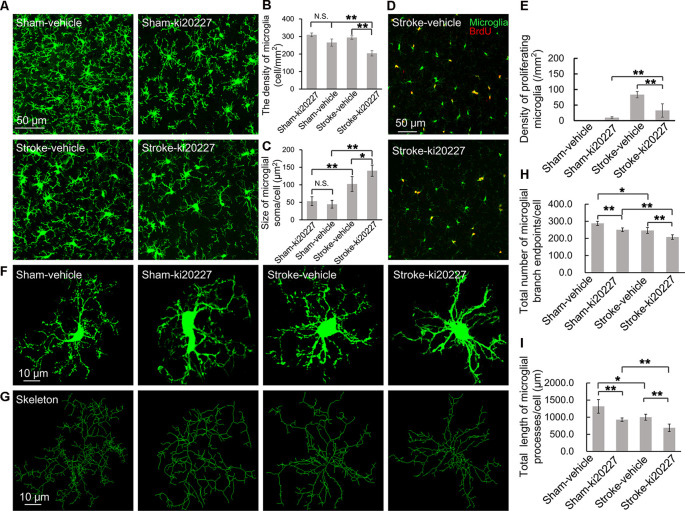
ki20227-induced morphological changes in the microglia following reperfusion after ischemia. **(A)** Representative confocal images of the microglia [*green*, green fluorescent protein (GFP)-positive microglia] in different groups. *Scale bar*, 50 μm. **(B)** Quantification of microglial density showing a significant difference between groups. *n* = 6 mice per group; N.S.: *p* < 0.05, **p < 0.01, *n* = 6. **(C)** Quantification of microglial soma size showing a significant difference between groups (*n* = 6 mice per group). N.S.: *p* < 0.05, *p < 0.05, **p < 0.01, *n* = 6. **(D)** Representative confocal images of microglial proliferation (*green*, GFP-positive microglia; *red*, BrdU-positive cells) in different groups. *Scale bar*, 50 μm. **(E)** Quantification of microglial proliferation showing a significant difference between groups (*n* = 6 mice per group). **p < 0.01, *n* = 6. **(F,G)** Representative confocal images of the microglia (*green*, GFP-positive microglia) and skeletonized microglia in the vehicle-treated sham, ki20227-treated sham, vehicle-treated stroke, and ki20227-treated stroke mice. The image sizes (*scale bars*) in panels **(F,G)** are of the same length. *Scale bar*, 10 μm. **(H,I)** Analysis of the total number of microglial branch endpoints and the total length of microglial processes in all groups. Note that there was a significant difference between the vehicle-treated and ki20227-treated groups. The total number of microglial branch endpoints and the total length of microglial processes were significantly decreased after treatment with ki20227 on day 3 after global ischemia. *p < 0.05, **p < 0.01, *n* = 6.

The effects of cerebral ischemia on the microglia were then evaluated. To clarify the response of the microglia after the inhibition of CSF1R, newly proliferated microglia in the somatosensory cortex were imaged, and the density and soma size of the microglia were also measured. We first assessed the effect of ki20227-induced inhibition of CSF1R in sham CX3CR1^GFP/+^ mice. Although the number of newly proliferated microglia was slightly increased in sham mice compared to control mice, the density and soma size of the microglia were not significantly changed after the inhibition of CSF1R by ki20227 (Figures 2A–E). We further assessed the effect of ki20227 on microglial activation after stroke. In the present study, confocal imaging indicated that the density of the microglia was significantly decreased in ki20227-treated stroke mice compared with vehicle-treated stroke mice 3 days after reperfusion [density (per square millimeter): vehicle-treated stroke mice, 294.3 ± 10.2, vs. ki20227-treated stroke mice, 204.6 ± 15.4; *p < 0.05; Figures 2A,B]. In addition, there was a significant change in microglial soma size between the ki20227-treated and vehicle-treated stroke mice on day 3 after reperfusion [soma size (per square micrometer): vehicle-treated stroke mice, 102.3 ± 20.8, vs. ki20227-treated stroke mice, 140.3 ± 15.9; *p < 0.05; Figure 2C]. Furthermore, there were significantly fewer newly proliferated microglia in the ki20227-treated than in vehicle-treated stroke mice on day 3 after reperfusion (number of proliferated microglia: vehicle-treated stroke mice, 93.5 ± 10.1, vs. ki20227-treated stroke mice, 32.8 ± 21.4; **p < 0.01; Figures 2D,E).

To precisely investigate how the microglia from CX3CR1^GFP/+^ mice respond to global ischemia following ki20227 treatment at the morphological level, ImageJ software was used to quantitatively analyze the skeletonized microglial morphology. All images of the microglia were collected from the somatosensory cortex. In the sham groups, we summarized the length of the microglial processes and the number of branch points in the vehicle-treated and the ki20227-treated sham groups. There was a significant difference in the number of branch endpoints, but no difference in the total process length of individual microglia between the vehicle-treated and ki20227-treated sham groups. We also found that there was a significant difference in the total process length of individual microglia between the vehicle-treated and ki20227-treated sham groups. We further evaluated the effect of ki20227 on microglial morphology in ischemic mice. In mice not treated with the antagonist, the total number of microglial branch endpoints and the total process length of each microglia were significantly reduced by global ischemia after 3 days of reperfusion. Remarkably, we found significant reductions in the number of microglial total branch endpoints and the process length in the ki20227-treated stroke group compared to the vehicle-treated stroke group on day 3 after reperfusion [total number of microglial branch endpoints/cell: vehicle-treated stroke group, 246.3 ± 13.7, vs. ki20227-treated stroke group, 207.8 ± 13.3; total length of microglial processes/cell (in micrometers): vehicle-treated stroke group, 999.6 ± 86.6, vs. ki20227-treated stroke group, 688.7 ± 110.6; **p < 0.01; Figures 2F–I].

### Inflammation Is Induced by Ischemia Combined With ki20227 Treatment

**Figure 3:**
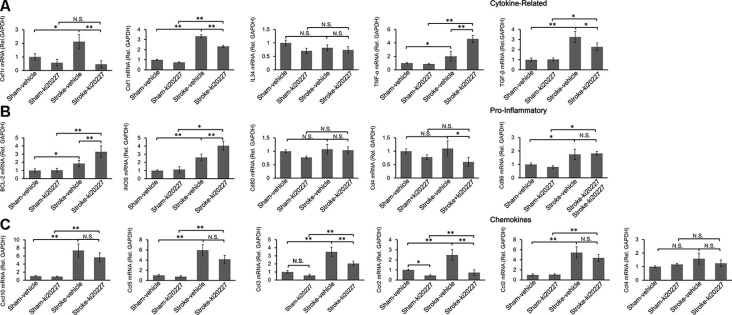
Response of the relative mRNA expressions of various inflammatory gene markers to ki20227 treatment. **(A)** Cytokines-related genes. **(B)** Pro-inflammatory genes. **(C)** Chemokines. There was little difference in the mRNA expression levels of inflammatory cytokines between the vehicle-treated and ki20227-treated sham mice on day 3. However, the mRNA expression levels of the cytokine-related genes, pro-inflammatory genes, and chemokines were significantly different between the ki20227-treated and vehicle-treated stroke mice on day 3 after reperfusion (*n* = 6 mice per group). N.S.: *p* > 0.05, *p < 0.05, **p < 0.01.

To determine whether inflammation is involved in the effect of ki20227 treatment, TGF-β, CSF1R, CSF1, CXCL10, BCL-2, iNOS, CCL5, TGF-α, IL34, CD86, CD80, CCR2, CCL2, CCL3, CCL4, and CD4 were detected in ki20227-treated sham mice. The expression levels were barely changed in the ki20227-treated sham mice compared to the vehicle-treated sham mice. However, the expressions of several of the above-mentioned genes were significantly increased in vehicle-treated stroke mice compared to vehicle-treated sham mice 3 days after reperfusion. We found that the expression levels of some chemokines and cytokine receptors, such as CSF1R, CSF1, CCL3, CD4, and CCR2, were significantly different in ki20227-treated stroke mice compared to vehicle-treated stroke mice (Figures 3A,C). Furthermore, in the ki20227-treated stroke mice, the mRNA expression levels of pro-inflammatory factors such as iNOS and BCL-2 (Figure 3B) and the cytokine-related gene TNF-α were significantly increased compared with those in the vehicle-treated stroke mice on day 3 after reperfusion (Figure 3A), but the mRNA expression levels of anti-inflammatory factors such as TGF-β were significantly decreased in the ki20227-treated stroke mice compared with the vehicle-treated stroke mice on day 3 after reperfusion (Figure 3A). Additionally, quantitative analysis showed no marked difference in the mRNA expressions of other chemokines and inflammatory genes, such as CD80, IL34, CD86, CXCL10, CCL2, CCL4, and CCL5, between the vehicle-treated and ki20227-treated stroke mice on day 3 (Figures 3B,C).

### CSF1R Inhibition Promotes the Degeneration of Neurons During the Progression of Global Ischemia

**Figure 4:**
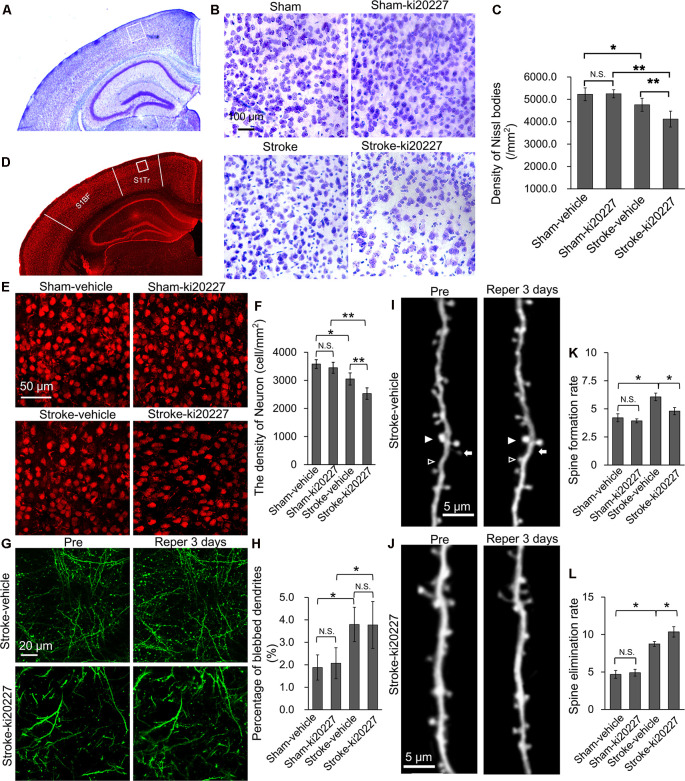
ki20227-induced neuronal degeneration in the cerebral cortex following reperfusion after transient global cerebral ischemia. **(A,B)** Representative image of Nissl bodies in the vehicle-treated sham, ki20227-treated sham, vehicle-treated stroke, and ki20227-treated stroke mice. The region of interest (ROI) is indicated by the *white box*. High-magnification images in panel **(B)** showing Nissl bodies in different groups. *Scale bar*, 100 μm. **(C)** Analysis of Nissl bodies in the vehicle-treated sham, ki20227-treated sham, vehicle-treated stroke, and ki20227-treated stroke mice. There was no difference between the vehicle-treated and ki20227-treated sham mice, but a significant difference was found between the vehicle-treated and ki20227-treated stroke mice (*n* = 6). *p < 0.05, **p < 0.01. **(D,E)** Confocal images showing neurons (*red*) of the somatosensory cortex in different groups. The ROI is indicated by the *white box*. High-magnification images in panel **(E)** show neurons in the different groups. *S1Tr*, primary somatosensory cortex, trunk region; *S1BF*, primary somatosensory cortex, barrel field. *Scale bar*, 50 μm. **(F)** Analysis of the density of neuron in the vehicle-treated sham, ki20227-treated sham, vehicle-treated stroke, and ki20227-treated stroke mice. Significant differences were found between the vehicle-treated and ki20227-treated stroke mice (*n* = 6). N.S: *p* < 0.05, *p < 0.05, **p < 0.01. **(G)** ki20227 did not affect the reversible recovery of the dendritic structure (*green*) following global cerebral ischemia–reperfusion. Morphological changes in dendritic structures before ischemia and 3 days after reperfusion. *Scale bar*, 20 μm. **(H)** Quantification of the percentage of blebbed dendritic structures in vehicle-treated sham, ki20227-treated sham, vehicle-treated stroke, and ki20227-treated stroke mice. Note that there was a significant difference in dendritic beading between sham and stroke mice (*n* = 6). N.S.: *p* < 0.05, *p < 0.05. **(I,J)** *In vivo* imaging of the dendritic structure in the different groups. The *open arrowheads*, *arrows*, and *filled arrowheads* indicate newly formed spines, eliminated spines, and stable spines, respectively. *Scale bar*, 5 μm. **(K,L)** The spine formation rate and spine elimination rate were significantly different between groups (*n* = 6 mice per group). *p < 0.05, **p < 0.01.

To determine the effect of ki20227 on the degeneration of neurons, the density of Nissl bodies was analyzed after Nissl staining (Figures 4A,B). In the ki20227-treated sham group, the density of Nissl bodies was not significantly different from that in the vehicle-treated sham group. However, in the ki20227-treated stroke mice, the density of Nissl bodies was significantly lower than that in the vehicle-treated stroke mice on day 3 after reperfusion [density (per square millimeter): vehicle-treated stroke mice, 4,756.8 ± 292.1, vs. ki20227-treated stroke mice, 4,116.9 ± 355.4; **p < 0.01; Figure 4C]. We assessed the effect of ki20227-induced inhibition of CSF1R on the density of neurons (Figures 4D,E). There was no significant difference in the density of neurons in sham mice before and after inhibition of CSF1R by ki20227. However, the density of neurons was significantly decreased in the ki20227-treated stroke mice compared with the vehicle-treated stroke mice on day 3 after reperfusion [density (per square millimeter): vehicle-treated stroke mice, 3,050.4 ± 216.7, vs. ki20227-treated stroke mice, 2,529.1 ± 204.8; *p < 0.05; Figure 4F].

We further examined whether ki20227 affects the recovery of the dendritic structure by evaluating the percentage of blebbed dendrites after stroke (Figure 4G). Time-lapse imaging revealed no significant dendritic structural damage in mice after ki20227 treatment, but a significant difference in damage between the vehicle-treated sham mice and the vehicle-treated stroke mice on day 3. Noteworthy is that there was a significant difference in damage between the ki20227-treated sham mice and ki20227-treated stroke mice on day 3. Furthermore, we examined the recovery of the dendritic structure in the vehicle-treated and ki20227-treated stroke mice and found no significant difference in the recovery of the dendritic structure on day 3 (percentage of blebbed dendrites: vehicle-treated stroke group, 3.8 ± 0.8, vs. ki20227-treated stroke group, 3.8 ± 1.0; *p* < 0.05, *n* = 6; Figure 4H). To further investigate the effect of ki20227 on dendritic spine plasticity after global cerebral ischemia, we assessed the formation and elimination of spines (Figures 4I,J). *In vivo* imaging showed that there were no significant differences in the spine elimination rate or formation rate between the vehicle-treated and ki20227-treated sham mice on day 3. However, when we further evaluated the formation and elimination rates of dendritic spines in ischemia mice, live imaging revealed that both the elimination rate and the formation rate were significantly higher in the vehicle-treated stroke mice than in the vehicle-treated sham mice on day 3 after reperfusion (Figures 4I,G). Importantly, the spine elimination rate in the ki20227-treated stroke mice was even higher than that in the vehicle-treated stroke mice, and the formation rate was significantly lower in the ki20227-treated than in vehicle-treated stroke mice on day 3 after reperfusion (before reperfusion: vehicle-treated stroke group: elimination rate, 8.74 ± 0.82%; formation rate, 6.06 ± 0.83%; ki20227-treated stroke group: elimination rate, 10.35 ± 1.68%; formation rate, 4.79 ± 0.64%; *p < 0.05, **p < 0.01, *n* = 6; Figures 4K,L).

### CSF1R Inhibition Prevents the Progression of Stroke Pathology

**Figure 5:**
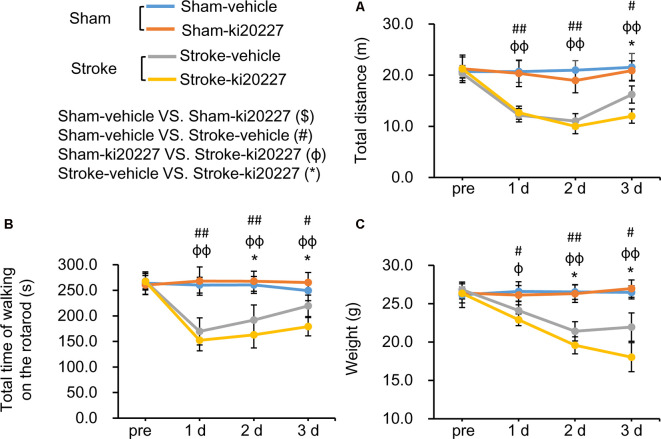
ki20227-induced behavioral deficits after global ischemia–reperfusion. **(A)** The tracks of the vehicle-treated sham, ki20227-treated sham, vehicle-treated stroke, and ki20227-treated stroke mice before and after the experiments. **(B)** There were significant differences in the total time spent on the rotarod between groups. **(C)** There were significant differences in mouse weight between groups. Vehicle-treated vs. ki20227-treated sham mice ($), vehicle-treated sham mice vs. vehicle-treated stroke mice (#), ki20227-treated sham mice vs. ki20227-treated stroke mice (φ), and vehicle-treated vs. ki20227-treated stroke mice (*). *p < 0.05, ^#^*p* < 0.05, ^##^*p* < 0.01, ^φ^*p* < 0.05, ^φφ^*p* < 0.01, *n* ≥ 6.

The open-field test and rotarod test were performed to evaluate behavioral deficits in stroke mice. There were no significant differences in the total distance traveled, total time spent on the rotarod, or weight between the vehicle-treated and ki20227-treated sham mice (Figures 5A–C). However, significant decreases in the total distance traveled, total time spent on the rotarod, and weight were detected in the vehicle-treated stroke mice compared with the vehicle-treated sham mice after reperfusion (Figures 5A–C). Importantly, we found that, in the ki20227-treated stroke mice, there were significant decreases in the total distance traveled, total time spent on the rotarod, and weight compared with those in the vehicle-treated stroke mice after reperfusion (Figures 5A–C).

## Discussion

The microglia are ramified and dynamic brain-resident myeloid cells (Mosher et al., 2012; Michell-Robinson et al., 2015), and the complexity of their morphology and function is determined by the physical conditions under which their dynamic ramified processes surveil the microenvironment and by the pathology driving their phagocytosis of pathogens or cellular debris (Tremblay et al., 2010; Fontainhas et al., 2011). Studies have elucidated the role of stimuli in altering microglial activity in models of CNS injury pathology (Fontainhas et al., 2011; Tremblay et al., 2011; Masuch et al., 2016). Our results show that treatment with the CSF1R kinase inhibitor ki20227 inhibits the proliferation of microglia and alters microglial morphology after ischemic stroke. Furthermore, these changes aggravate neurodegeneration and change the plasticity of neuronal dendrites after global cerebral ischemia.

De-ramification is one of the cellular morphological properties of activated microglia (Kreutzberg, 1996; Stence et al., 2001); the microglia can retract their processes and alter their gene expression profiles in response to ischemia (Khan et al., 2017; Ju et al., 2018). Such morphological alterations are closely related to the velocity of local blood flow. When blood flow is completely stopped, the mobility of microglial protrusions, including extension and retraction, is lost (Masuda et al., 2011). In addition to the altered morphology, modified gene expression is considered the main event in microglial activation. Such alterations, which include pro- and anti-inflammatory effects, are dependent on the specific stage of pathology (Bell et al., 2010; Denieffe et al., 2013). Based on these findings, our study aimed to investigate the effect of a predominant factor, CSF1R, which is a cytokine that participates in morphological changes in and proliferation of microglia, on the functional and phenotypical modification of the microglia during ischemia–reperfusion. CSF1R and its downstream pathway are closely related to the survival and maintenance of the microglia. PLX3397 is an efficient, selective, ATP-competitive inhibitor of CSF1R, and as a robust inhibitor, it can cause a dramatic reduction in the microglial population in the adult brain. However, unlike PLX3397, ki20227 is a highly selective c-Fms tyrosine kinase (CSF1R) inhibitor like GW2580. In adult mice, ki20227 has little effect on the survival rate of microglia, and GW2580 (75 mg/kg) has been used in the past without having a significant effect on the microglial survival rate. The role of the microglia after stroke is still controversial. Treatment with PLX3397 may cause a remarkable change in microglial cell density, while ki20227-induced inhibition of CSF1R does not induce a notable change in the number of microglia. In this study, we selected ki20227 to prevent the elimination of a large number of microglia due to CSF1R inhibition.

Used as an antagonist, ki20227 induced a marked microglial de-ramification in both sham and ischemic mice, suggesting that morphological changes in the microglia are closely related to the CSF1R cascade. Importantly, microglia proliferation and soma size are more obvious markers of activation; no changes in the microglial soma size and survival indicated that the microglia were not activated, although there were slight modifications in the microglial processes and microglia proliferation. In the sham group, ki20227, unlike other strong microglial inhibitors, did not cause the loss of a large number of microglia, but had a similar effect as GW2580, suggesting that the drug did not lead to microglial activation. Additionally, inflammatory cytokines, which are markers of activated microglia, were not significantly changed by ki20227 treatment. There results suggested that the drug did not lead to microglia activation in sham mice. Furthermore, ki20227 treatment resulted in a decline in the number of microglia and BrdU-labeled microglia in stroke mice. This result suggests that CSF1R inhibition reduced the number of newly proliferated microglia after global cerebral ischemia. Moreover, ki20227 treatment showed that a considerable number of microglial processes were retracted, suggesting that CSF1R inhibition induced excessive de-ramification of activated microglia after global cerebral ischemia. In addition to exerting surveillance functions, the microglia exhibit pro-inflammatory and neurotropic activities *via* increased expressions of cell-surface receptors and the release of cytokines and chemokines (Perego et al., 2011). During reperfusion, the increased release of microglial cytokines, chemokines, and other pro-inflammatory molecules in response to the ischemic stimuli suggested that these molecules play crucial roles in the activation, differentiation, regulation, and the functions of innate and adaptive immunity after stroke (Ramesh et al., 2013). These cytokine-related genes, such as TNF-α, iNOS, and TNF-β, which are pro- or anti-inflammatory, may contribute to the impairment of neuronal and brain damage after stroke (Lin et al., 2016). We observed a significant enhancement of the cytokine-related genes such as TNF-α and iNOS and the downregulated gene TNF-β in ischemic mice after ki20227 treatment, indicating that these cytokine-related genes may be a risk factor after stroke. Stroke caused an increased release of cytokines/chemokines such as CCL and its ligands MCP-1/CCL2, IP-10/CXCL10, and RANTES/CCL5. These cytokine/chemokine networks related to the activation of the microglia were reestablished after stroke, suggesting that ki20227 treatment further caused excessive immune response after ischemia–reperfusion. Furthermore, we found that stroke induced a high expression of CSF1R, which is closely related to microglial proliferation. CSF1R expression was significantly decreased after ki20227 treatment, suggesting that stroke-related microglial proliferation may be dependent on the CSF1R signaling pathway. In addition, as a marker of monocytes, increased CCR2 mRNA expression is indicative of monocyte infiltration after stroke. However, it was previously shown that CCR2 mRNA expression is inhibited by ki20227 (Ohno et al., 2008). Whether the infiltration of monocytes has a robust effect on neuronal structure remains to be evaluated in future studies.

In sham-operated mice, the neurons of the somatosensory cortex were not affected by ki20227 treatment. However, ki20227 treatment exacerbated neurodegeneration after stroke, which suggested that CSF1R is necessary for the recovery of neurons damaged by stroke. In addition, the reversible recovery of dendritic structures was not affected by ki20227 treatment during reperfusion, suggesting that CSF1R inhibition has less influence on the integrity of neural networks than does local blood flow (Zhu et al., 2017b). Although ki20227 treatment did not change the reversible recovery of dendritic structures after ischemia, our study showed that ki20227 treatment exacerbated the loss of dendritic spines when combined with ischemia, indicating that CSF1R inhibition changed the plasticity of the dendritic spine during ischemia. Whether ki20227 has a long-term effect on the stability of the neuronal structure and the plasticity of cortical circuits remains to be evaluated in future studies. Damage to neurons is a major manifestation of stroke in the CNS. An unstable neuronal structural and functional state is considered crucial for neural development and circuit plasticity under normal physical conditions (Grutzendler et al., 2002; Davalos et al., 2005; Zuo et al., 2005; Bhatt et al., 2009); however, this state can be changed by ischemia and other pathological insults (Hasbani et al., 2001; Heiss et al., 2017; Suresh and Dunaevsky, 2017; Zhu et al., 2017b). The unstable neuronal structural and functional state after ischemia is thought to be related to the rewiring of neuronal circuits and to contribute to the recovery of cortical function (Dijkhuizen et al., 2001; Ward and Cohen, 2004; Murphy and Corbett, 2009). In our present study, the behavioral tests showed that ki20227 treatment alone did not affect the behavior of healthy mice but that ki20227 treatment further reduced the activity of the mice after stroke, suggesting that CSF1R inhibition may have accumulative and detrimental effects on the restoration of behavioral ability following ischemia.

In summary, this study suggests that pharmacological inhibition of CSF1R by ki20227 causes excessive microglial de-ramification and inflammatory responses, induces a decrease in microglial proliferation, and aggravates neuronal degeneration, the loss of dendritic spines, and behavioral deficits after transient global cerebral ischemia. Therefore, inhibition of CSF1R in the microglia may have a negative clinical effect following ischemic stroke.

## Data Availability Statement

All datasets generated for this study are included in the article.

## Ethics Statement

This study was carried out in accordance with the regulations of Lanzhou University and the ARRIVE guidelines. All experimental procedures and protocols were approved by the Ethics Committee of Lanzhou University. Written informed consent was obtained from the owners for the participation of their animals in this study.

## Author Contributions

FJ, BH, and HR designed the study. FJ, BH, CJ, GW, ZW, DW, YK, JS, and PW performed the experiments. MZ and DW analyzed the data. FJ, BH, and CJ wrote the article.

## Conflict of Interest

The authors declare that the research was conducted in the absence of any commercial or financial relationships that could be construed as a potential conflict of interest.

## Footnotes

^1^http://rsb.info.nih.gov/ij/

^2^http://imagejdocu.tudor.lu/
